# The association of socioeconomic status on treatment strategy in patients with stage I and II breast cancer in the Netherlands

**DOI:** 10.1007/s10549-021-06308-2

**Published:** 2021-06-26

**Authors:** M. D. Filipe, S. Siesling, M. R. Vriens, P. van Diest, A. J. Witkamp

**Affiliations:** 1 Department of Surgery, Cancer Centre, University Medical Centre Utrecht, PO Box 85500, 3508 GA Utrecht, The Netherlands; 2 Department of Research and Development, Netherlands Comprehensive Cancer Organisation, Utrecht, The Netherlands; 3 Department of Health Technology and Services Research, Technical Medical Centre, University of Twente, Enschede, The Netherlands; 4 Department of Pathology, University Medical Centre Utrecht, Utrecht, The Netherlands

**Keywords:** Socioeconomic status, Cancer registry, Population-based study

## Abstract

**Background:**

Previous studies have shown that socioeconomic status (SES) influences breast cancer therapy. However, these studies were performed in countries with unequal access to healthcare. Therefore, the aim of this study is to investigate whether SES also contributes to the likelihood of receiving a certain therapy in the Netherlands, a country with supposedly equal access to healthcare.

**Materials and methods:**

From the Netherlands Cancer Registry, 105,287 patients with newly diagnosed stage I or II breast cancer diagnosed between 2011 and 2018 were selected for analysis. SES was calculated from the average incomes of each postal code, which were divided into 10 deciles. Primary outcome was the effect of SES on the likelihood of undergoing surgery and secondary outcome was the effect of SES on the likelihood of the type of surgery. Both outcomes were corrected for patient, tumor, and hospital characteristics and were expressed as odds ratio (OR) with 95% confidence interval (CI).

**Results:**

SES did not affect the likelihood of a breast cancer patient to undergo surgery (OR 1.00 per 10% stratum). In contrast, increased age and higher tumor stage were the most important factors determining whether patients underwent surgery.

Patients with higher SES were less likely to undergo mastectomy (OR 0.98). Additionally, more recently diagnosed patients were less likely to undergo mastectomy (OR 0.93 per year) while patients with higher tumor stage were more likely to undergo mastectomy (OR 3.42).

**Conclusion:**

SES does not affect whether a patient undergoes surgery; however, higher SES increased the likelihood of BCT.

## Introduction

Breast cancer is the most common cancer in women and the second most common cause of death due to cancer in women worldwide [1]. There are roughly 17,000 new cases of breast cancer in the Netherlands every year. Additionally, over 3000 people of the Dutch population die annually due to breast cancer [2, 3].

Surgical resection of the primary tumor is the treatment of choice in patients with newly diagnosed breast cancer. Tumor stage and molecular characteristics determine the type of surgery. The main types of surgery for stages I and II are mastectomy and breast-conserving therapy (BCT) [4–6]. In the Netherlands, the percentage BCT is about 65% and this has been shown to differ between regions [7]. Reasons for these differences can be the preference of the clinician, age of patient, tumor grade, tumor stage, and hormone receptor status [7]. Moreover, socioeconomic status (SES) might be of influence here since it is not equally spread over the country [8].

SES is a complex classification system to stratify economic and social factors [9]. SES has shown to be of influence the incidence and severity of diseases. Low SES is associated with a higher incidence of lifestyle related risk factors, such as smoking, higher BMI, and drug use [10]. This high risk behavior leads to an increased risk for the development of disease, such as diabetes, cardiovascular disease, psychiatric disorders, and numerous types of cancer [10–14].

Differences in treatment between SES classes have been described in a systematic review in which patients with breast cancer who, among other factors, had a higher SES were more likely to undergo BCT [15]. Additionally, in the United States of America unequal access to healthcare due to financial barriers leads to therapeutic choices based on income [16]. Various studies in the United States showed that SES influences the choice for the surgical procedure [17, 18]. Furthermore, a Danish study showed that low SES stage I or II breast cancer patients tended to have more mastectomies despite equal access to healthcare. There was no clear explanation for this disparity [19]. However, this was not a population-based cohort, and the study cohort was closed 1998 while treatment options have changed since then.

In the Netherlands, there is universal healthcare which means that citizens do not have financial barriers when requesting medical attention [16]. Additionally, the compulsory insurance covers almost all costs for hospitals and primary care [16, 20].

Currently, no studies have analyzed the influence of SES on treatment choices and type of surgery in patients with stage I and II breast cancer where there are no financial barriers to healthcare. The aim of this study was therefore to determine whether SES influences the treatment of stage I and II breast cancer in the Netherlands since there are no barriers for access to healthcare.

## Materials and methods

### Study design and population

In this nationwide population-based study, we selected breast cancer patients of the Netherlands Cancer Registry (NCR). The present study focused on primary stage I and II breast cancer patients treated between January 1st, 2011 and December 31st, 2018. Only new-onset breast cancer patients were included in this study.

### Definitions

The NCR contains patient, tumor, and treatment characteristics. Tumors are categorized according to the tumor, node, and metastasis (TNM) classification system [21]. Due to changes in the N1 category from the 5th to the 6th editions of the International Classification of Diseases for Oncology, we classified the number of positive lymph into N categories. Patients without lymph node involvement were classified as N0 and patients with 1 to 3 positive lymph nodes were classified as N1. TNM was converted to tumor stage (stage I or stage II). Histological subtype consisted of lobular, ductal, mucinous, medullary tubular, or not specified [22]. Tumor grade was divided into low grade, intermediate grade, and high grade [23].

SES was determined using the average income of a household according to the four-digit postal code in the Netherlands at time of diagnosis and surgical procedure, and was defined according to the Dutch Bureau of Statistics (CBS) [24]. Furthermore, the average incomes of each postal code were divided into 10 deciles. Additionally, hospital volume was stratified based on the number of breast cancer patients treated per year: low volume (< 100), medium volume (100–149), and high volume (> 150), as described in previous studies [25].

### Outcomes

Primary outcome was the effect of SES on the likelihood of a new-onset breast cancer patients undergoing surgery versus no surgical treatment. Secondary outcome was determining the effect of SES on type of surgery (BCT or mastectomy). Both outcomes were determined after correcting for patient, tumor, and hospital characteristics.

### Statistics

Descriptive statistics were used to describe patient, tumor, and treatment characteristics. Continuous data were described with mean along with standard deviation (SD), or with median and interquartile range (IQR), depending on whether or not the data were normally distributed. Mann–Whitney *U* tests or Student’s *t* tests were used to test differences between groups of not normally and normally distributed continuous data, respectively. Differences between categorical data were analyzed with Chi-Square or Fisher’s exact tests.

Since some data were missing during the study period, multiple imputation by chained equations (MICE) was performed using the *MICE* package in R. After comparing and correlating the missing to the non-missing data, it was concluded that the values were missing at random. The imputation was repeated 20 times, followed by application of Rubin's rule to combine parameter estimates and standard errors [26, 27]. Imputed data were later compared to the complete cases to determine validity of the imputation model. Subsequently, the imputed data were used for analyses.

Multivariable regression analyses were performed to study the association between SES and the likelihood [quantified in odds ratio (OR) and 95% confidence interval (CI)] of undergoing a certain treatment strategy (no surgery vs. surgery, and BCT vs. mastectomy) in patients with stage I or II breast cancer. Possible confounding factors and effect modifiers considered were age at diagnosis, stage (1 or 2) and co-morbidities. Two-sided P values below 0.05 were considered statistically significant.

All calculations were performed using RStudio 1.2.5001 (with R version: × 64 3.6.3). Visualization of plots was performed using the *ggplot2* package.

## Results

Between 2011 and 2018, 105,287 patients had new-onset stage I or II breast cancer, of whom 6840 patients (6.5%) did not undergo surgery. Furthermore, 98,447 stage I or II breast cancer patients underwent surgery of whom 65,888 patients underwent BCT and 32,559 patients had undergone mastectomy suitable for analysis.

**Fig. 1:**
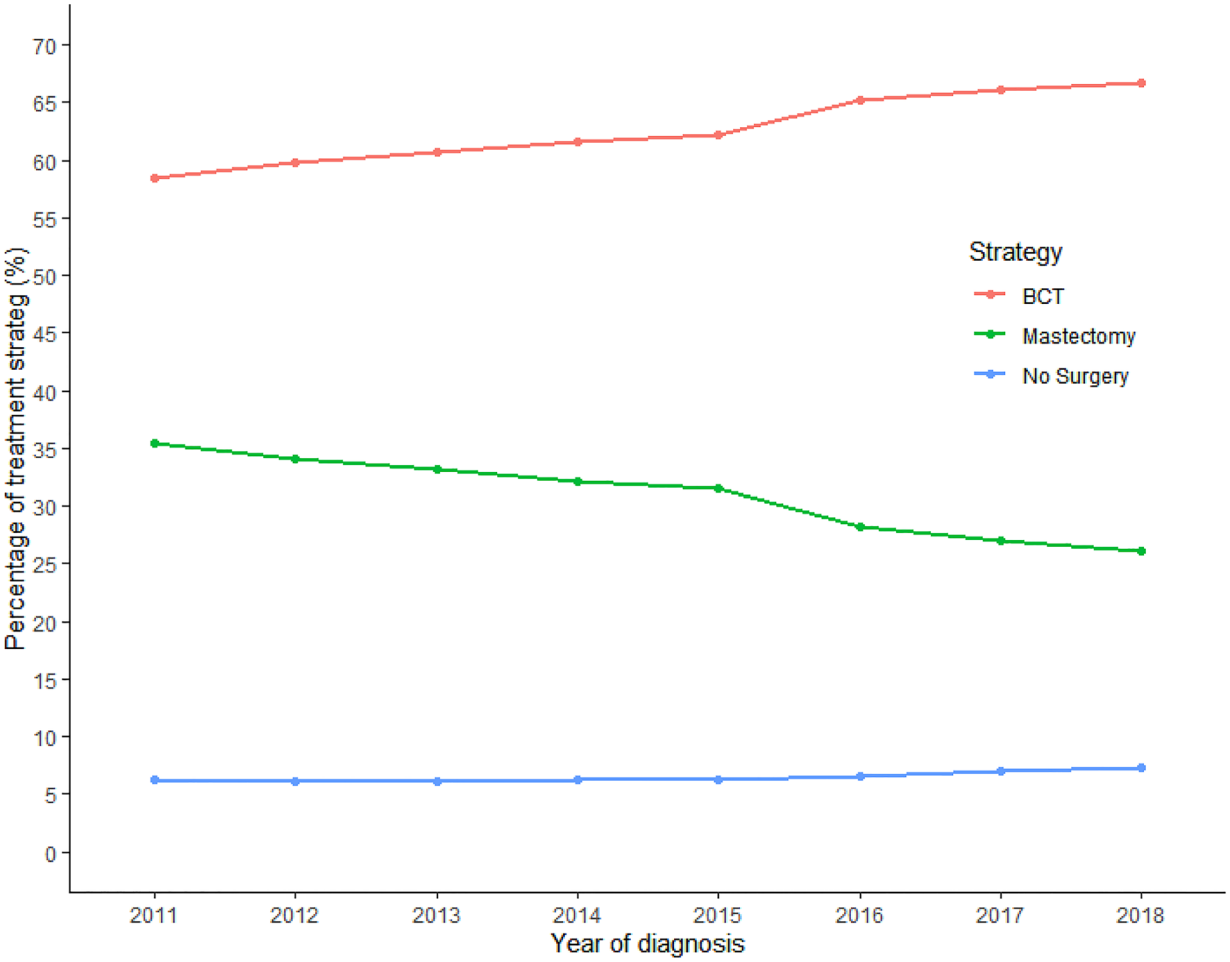
Trends of treatment strategy of new-onset stage I and stage II breast cancer over time. *BCT* breast-conserving therapy

Table 1 shows the baseline characteristics of all 105,287 stage I or II breast cancer patients suitable for analysis. Mean age was 62.0 years. The national screening program detected breast cancer in 39,094 (37.7%) patients and 393 (0.4%) patients had a positive oncological history other than breast cancer. A total of 98,447 (93.5%) stage I or II breast cancer patients underwent surgery (BCT or mastectomy), while 6840 (6.5%) breast cancer patients had no surgery but were treated with only chemotherapy, hormone therapy, and/or radiotherapy. The proportion of breast cancer patients undergoing non-surgical treatment slightly increased over time (Fig. 1). Stage I or II breast cancer was evenly spread among the different strata of SES. The proportion of BCT substantially increases, while the proportion mastectomies decreases.

**Table 1:** Baseline characteristics of all new-onset breast cancer patients diagnosed in the Netherlands between 2011 and 2018

Characteristic	*N* = 105,287
Age in years, mean (SD)	62.0 (13.5)
Age groups	
Under 40 years, *N* (%)	4494 (4.3%)
40–50 years, *N* (%)	15,584 (14.9%)
50–75 years, *N* (%)	66,735 (63.6%)
Over 75 years, *N* (%)	18,106 (17.3%)
Affected side	
Left, *N* (%)	53,379 (50.9%)
Right, *N* (%)	51,540 (49.1%)
Medical history	
No medical history, *N* (%)	95,874 (92.9%)
Positive non-oncological medical history, *N* (%)	6885 (6.7%)
Positive oncological medical history, *N* (%)	393 (0.4%)
Detected by national screening program, *N* (%)	39,094 (37.7%)
Type of treatment	
No surgery, *N* (%)	6805 (6.5%)
BCT, *N* (%)	65,704 (62.6%)
Mastectomy, *N* (%)	32,410 (30.9%)
Tumor stage	
Stage I, *N* (%)	61,011 (58.2%)
Stage II, *N* (%)	43,908 (41.8%)
Socioeconomic status	
0–9%, *N* (%)	10,349 (9.9%)
10–20%, *N* (%)	10,428 (9.9%)
20–30%, *N* (%)	10,274 (9.8%)
30–40%, *N* (%)	10,289 (9.8%)
40–50%, *N* (%)	10,557 (10.1%)
50–60%, *N* (%)	10,233 (9.8%)
60–70%, *N* (%)	10,278 (9.8%)
70–80%, *N* (%)	10,616 (10.1%)
80–90%, *N* (%)	10,760 (10.3%)
90–100%, *N* (%)	11,135 (10.6%)

*SD* standard deviation, *N* number, *BIRADS* breast imaging reporting and data system, *BCT* breast-conserving therapy

Table 2 shows the different characteristics of patients stratified for surgery-or-not. Patients not undergoing surgery were significantly older, and more often of higher stage and grade, and more often had HER2-negative and estrogen receptor-positive tumors. Furthermore, patients undergoing surgery were more often triple negative (3.8%) compared to patients not undergoing surgery (1.8%). 9465 (91.2%) of the patients with the lowest SES underwent surgery which was significantly less than the 10,590 (94.7%) of the patients with the highest SES. However, after correcting for patient and tumor characteristics in multivariable analysis, SES was no predictor for undergoing surgery, while age, triple-negative receptor status, and tumor stage (highest absolute z value) played the most important role in determining surgery-or-not (Table 3).

**Table 2:** Differences between no surgery and surgery of new-onset breast cancer patients

Parameter	No surgery *n* = 6,840	Surgery *n* = 98,447	*p* value
Age in years, mean (SD)	82.1 (11.1)	60.6 (12.6)	< 0.001
Age groups			< 0.001
Under 40 years, *N* (%)	50 (1.1%)	4476 (98.9%)	
40–50 years, *N* (%)	139 (0.9%)	15,538 (99.1%)	
50–75 years, *N* (%)	907 (1.4%)	66,013 (98.6%)	
Over 75 years, *N* (%)	5744 (31.6%)	12,420 (68.4%)	
Detected during screening, *N* (%)	233 (3.4%)	39,370 (40.0%)	< 0.001
Socioeconomic status			< 0.001
0–9%, *N* (%)	917 (8.8%)	9465 (91.2%)	
10–20%, *N* (%)	774 (7.4%)	9700 (92.6%)	
20–30%, *N* (%)	717 (7.0%)	9588 (93.0%)	
30–40%, *N* (%)	665 (6.4%)	9662 (93.6%)	
40–50%, *N* (%)	675 (6.4%)	9912 (93.6%)	
50–60%, *N* (%)	685 (6.7%)	9587 (93.3%)	
60–70%, *N* (%)	623 (6.0%)	9688 (94.0%)	
70–80%, *N* (%)	594 (5.6%)	10,056 (94.4%)	
80–90%, *N* (%)	595 (5.5%)	10,199 (94.5%)	
90–100%, *N* (%)	595 (5.3%)	10,590 (94.7%)	
Tumor stage			< 0.001
Stage I, *N* (%)	2394 (3.9%)	58,617 (96.1%)	
Stage II, *N* (%)	4446 (10.0%)	39,830 (90.0%)	
Medical history			< 0.001
No medical history, *N* (%)	6562 (2.6%)	91,299 (97.4%)	
Positive non-oncological medical history, *N* (%)	247 (3.5%)	6774 (96.5%)	
Positive oncological medical history, *N* (%)	31 (0.5%)	374 (92.3%)	
Hormone receptor status			
Her2 receptor negative, *N* (%)	6331(6.8%)	86,808 (93.2%)	< 0.001
Progesterone receptor positive, *N* (%)	4636(6.5%)	66,854 (93.5%)	0.831
Estrogen receptor positive, *N* (%)	5820(6.7%)	81,244 (93.3%)	< 0.001
Triple negative	128(3.3%)	3757 (96.7%)	< 0.001
Tumor grade			< 0.001
Low grade, *N* (%)	1910 (7.2%)	24,757(92.8%)	
Intermediate grade, *N* (%)	3505 (6.9%)	47,590(93.1%)	
High grade, *N* (%)	1425 (5.2%)	26,100(94.8%)	
Histological tumor type			< 0.001
No special type, *N* (%)	5198 (6.1%)	79,460 (93.9%)	
Lobular (ILC), *N* (%)	1072 (8.5%)	11,536 (91.5%)	
Both, *N* (%)	91 (2.9%)	3041 (97.1%)	
Mucinous, *N* (%)	300 (14.6%)	1756 (85.4%)	
Medullary, *N* (%)	1(0.2%)	594 (99.8%)	
Tubular, *N* (%)	18(2%)	898 (98.0%)	
Other, *N* (%)	160 (12.1%)	1162(87.9%)	
Hospital volume			0.395
Low volume	662 (6.7%)	9166 (93.3%)	
Average volume	1649 (6.4%)	24,302 (93.6%)	
High volume	4529 (6.5%)	64,979 (93.5%)	
Year of diagnosis			< 0.001
2011, *N* (%)	792 (6.2%)	11,908 (93.8%)	
2012, *N* (%)	798 (6.1%)	12,180 (93.9%)	
2013, *N* (%)	800 (6.1%)	12,290 (93.9%)	
2014, *N* (%)	833 (6.3%)	12,332 (93.7%)	
2015, *N* (%)	815 (6.2%)	12,329 (93.8%)	
2016, *N* (%)	870 (6.6%)	12,324 (93.4%)	
2017, *N* (%)	961 (7.0%)	12,700 (93.0%)	
2018, *N* (%)	971 (7.3%)	12,384 (92.7%)	

*BCT* breast-conserving therapy, *N* number, *SD* standard deviation, *BIRADS* breast imaging reporting and data system, *ILS* invasive lobular carcinoma

**Table 3:** Multivariate regression analysis factors influencing non-surgical therapy versus surgery

Parameter	Estimate (β)	OR (95% CI)	Standard error	*Z* value	*p* value
SES (per 10% stratum)	0.010	1.01 (1.00–1.02)	0.005	1.877	0.060
Age (years)	− 0.169	0.84 (0.84–0.85)	0.002	− 94.497	< 0.001
Year of treatment	− 0.026	0.97 (0.96–0.99)	0.007	− 3.953	< 0.001
Hospital volume					
Small volume	NA	1.00 (reference)	NA	NA	NA
Average volume	0.010	1.01 (0.90–1.13)	0.058	0.172	0.863
Large volume	− 0.101	0.90 (0.82–1.00)	0.053	− 1.916	0.055
Tumor grade					
Low grade	NA	1.00 (reference)	NA	NA	NA
Intermediate grade	0.092	1.1 (1.02–1.18)	0.038	2.439	0.015
High grade	0.375	1.45 (1.32–1.60)	0.049	7.700	< 0.001
Histological subtype					
Ductal carcinoma	NA	1.00 (reference)	NA	NA	NA
ILC	− 0.031	0.97 (0.89–1.05)	0.043	− 0.723	0.470
Both	0.697	2.01 (1.59–2.53)	0.119	5.859	< 0.001
Mucinous	0.232	1.26 (1.08–1.47)	0.078	2.964	0.003
Medullary	3.040	20.89 (2.92–149.49)	1.004	3.028	0.002
Tubular	0.400	1.49 (0.90–2.47)	0.257	1.556	0.120
Other	0.027	1.03 (0.83–1.27)	0.107	0.254	0.799
Hormone receptor status					
Her2 receptor	− 0.136	0.87 (0.78–0.98)	0.057	− 2.376	0.017
Progesterone receptor positive	0.060	1.06 (0.98–1.15)	0.040	1.506	0.132
Estrogen receptor positive	− 0.063	0.94 (0.84–1.05)	0.054	− 1.148	0.251
Triple negative	0.678	1.97 (1.53–2.54)	0.129	5.25	< 0.001
Patient history					
No history of disease	NA	1.00 (reference)	NA	NA	NA
Non-oncological history	0.354	1.42 (1.23–1.64)	0.073	4.824	< 0.001
Oncological history	0.086	1.09 (0.72–1.64)	0.21	0.409	0.683
Tumor stage					
Stage I tumor	NA	1.00 (reference)	NA	NA	NA
Stage II tumor	− 0.609	0.54 (0.51–0.58)	0.032	− 18.999	< 0.001

*OR* odds ratio, *CI* confidence interval, *SES* socioeconomic status, *ILC* invasive lobular carcinoma, *NA* not applicable, *NAN* not a number

Stratified by type of breast cancer surgery (Table 4) patients who underwent mastectomy were slightly, but significantly, older and had lower SES. Additionally, patients with lower tumor stage more often received BCT. Furthermore, patients undergoing mastectomy were more often HER2-negative and estrogen receptor-positive compared to BCT counterparts. Moreover, increasing tumor grade and triple-negative receptor status was associated to an increased likelihood of undergoing mastectomy. After correcting for patient and tumor characteristics, SES remained a significant predictor for type of surgery where breast cancer patients with higher SES were significantly more likely to undergo BCT (Table 5). Breast cancer patients with the lowest SES stratum have an OR of 0.81 (or 44.9% less likely) of undergoing BCT compared to the highest SES stratum.

**Table 4:** Differences between BCT and mastectomy of new-onset breast cancer patients

	BCT *n* = 65,888	Mastectomy *n* = 32,559	*p* value
Age in years, mean (SD)	60.4 (11.3)	60.7 (14.7)	0.001
Age groups			
Under 40 years, *N* (%)	2155 (48.1%)	2321 (51.9%)	
40–50 years, *N* (%)	9510 (61.2%)	6028 (38.8%)	
50–75 years, *N* (%)	48,393 (73.3%)	17,620 (26.7%)	
Over 75 years, *N* (%)	5830 (46.9%)	6590 (53.1%)	
Socioeconomic status			< 0.001
0–9%, *N* (%)	6067 (64.1%)	3398 (35.9%)	
10–20%, *N* (%)	6290 (64.8%)	3410 (35.2%)	
20–30%, *N* (%)	6270 (65.4%)	3318 (34.6%)	
30–40%, *N* (%)	6448 (66.7%)	3214 (33.3%)	
40–50%, *N* (%)	6689 (67.5%)	3223 (32.5%)	
50–60%, *N* (%)	6466 (67.4%)	3121 (32.6%)	
60–70%, *N* (%)	6558 (67.7%)	3130 (32.3%)	
70–80%, *N* (%)	6840 (68.0%)	3216 (32.0%)	
80–90%, *N* (%)	7075 (69.4%)	3124 (30.6%)	
90–100%, *N* (%)	7185 (67.8%)	3405 (32.2%)	
Tumor stage			< 0.001
Stage I, *N* (%)	45,920 (78.3%)	12,697 (21.7%)	
Stage II, *N* (%)	19,968 (50.1%)	19,862 (49.9%)	
Medical history			< 0.001
No medical history, *N* (%)	61,375 (67.3%)	29,766 (32.7%)	
Positive non-oncological medical history, *N* (%)	4372 (63.2%)	2546 (36.8%)	
Positive oncological medical history, *N* (%)	141 (36.3%)	247 (63.7%)	
Hormone receptor status			
Her2receptor negative, *N* (%)	59,057 (67.8%)	28,008 (32.2%)	< 0.001
Progesterone receptor positive, *N* (%)	45,924 (68.4%)	21,265 (31.6%)	< 0.001
Estrogen receptor positive, *N* (%)	55,295 (68.0%)	26,028 (32.0%)	< 0.001
Triple negative, *N* (%)	2076 (55.3%)	1681 (44.7%)	< 0.001
Tumor grade			< 0.001
Low grade, *N* (%)	18,762 (75.7%)	6014 (24.3%)	
Intermediate grade, *N* (%)	31,109 (65.5%)	16,415 (34.5%)	
High grade, *N* (%)	16,017 (61.3%)	10,130 (38.7%)	
Histological tumor type			< 0.001
No special type, *N* (%)	55,100 (69.3%)	24,360 (30.7%)	
ILC, *N* (%)	6192 (53.7%)	5344 (46.3%)	
Both, *N* (%)	1560 (51.3%)	1481 (48.7%)	
Mucinous, *N* (%)	1200 (68.3%)	556 (31.7%)	
Medullary, *N* (%)	399 (67.2%)	195 (32.8%)	
Tubular, *N* (%)	750 (83.5%)	148 (16.5%)	
Other, *N* (%)	687 (59.1%)	475 (40.9%)	
Neo-adjuvant therapy, *N* (%)	7940 (61.9%)	4893 (38.1%)	< 0.001
Adjuvant therapy, *N* (%)	64,576 (77%)	19,337 (23%)	< 0.001
Detected during screening	31,574 (47.9%)	7796 (23.9%)	< 0.001
Hospital volume			< 0.001
Low volume	5825 (63.6%)	3341 (36.4%)	
Average volume	15,876 (65.3%)	8426 (34.7%)	
High volume	44,187 (68%)	20,792 (32%)	
Year of diagnosis			< 0.001
2011, *N* (%)	7411 (62.2%)	4497 (37.8%)	
2012, *N* (%)	7754 (63.7%)	4426 (36.3%)	
2013, *N* (%)	7943 (64.6%)	4347 (35.4%)	
2014, *N* (%)	8103 (65.7%)	4229 (34.3%)	
2015, *N* (%)	8178 (66.3%)	4151 (33.7%)	
2016, *N* (%)	8602 (69.8%)	3722 (30.2%)	
2017, *N* (%)	9009 (70.9%)	3691 (29.1%)	
2018, *N* (%)	8888 (71.8%)	3496 (28.2%)	

*BCT* breast conserving therapy, *N* number, *SD* standard deviation, *ILC* invasive lobular carcinoma

**Table 5:** Multivariate regression analysis factors influencing the likelihood of undergoing mastectomy compared BCT

Parameter	Estimate (β)	OR (95% CI)	Standard error	*Z* value	*p* value
SES (per 10% stratum)	− 0.023	0.98 (0.97–0.98)	0.003	− 9.013	< 0.001
Age (years)	0.004	1.00 (1.00–1.01)	0.001	7.758	< 0.001
Year of surgery	− 0.073	0.93 (0.92–0.94)	0.003	− 22.659	< 0.001
Hospital volume					
Small volume	NA	1.00 (reference)	NA	NA	NA
Average volume	0.009	1.01 (0.96–1.06)	0.027	0.323	0.747
Large volume	− 0.111	0.90 (0.85–0.94)	0.025	− 4.415	< 0.001
Tumor grade					
Low grade	NA	1.00 (reference)	NA	NA	NA
Intermediate grade	0.190	1.21 (1.16–1.26)	0.019	9.885	< 0.001
High grade	0.270	1.31 (1.25–1.37)	0.023	11.649	< 0.001
Histological subtype					
Ductal carcinoma	NA	1.00 (reference)	NA	NA	NA
ILC	0.625	1.87 (1.79–1.95)	0.022	28.307	< 0.001
Both	0.836	2.31 (2.13–2.49)	0.039	21.172	< 0.001
Mucinous	0.07	1.07 (0.96–1.19)	0.055	1.271	0.204
Medullary	− 0.055	0.95 (0.79–1.14)	0.093	− 0.588	0.556
Tubular	− 0.193	0.82 (0.69–0.99)	0.093	− 2.083	0.037
Other	0.271	1.31 (1.16–1.49)	0.064	4.217	< 0.001
Hormone receptor status					
Her2 receptor negative	− 0.128	0.88 (0.83–0.93)	0.027	− 4.775	< 0.001
Progesterone receptor positive	0.019	1.02 (0.98–1.06)	0.020	0.928	0.353
Estrogen receptor positive	0.002	1.00 (0.95–1.06)	0.028	0.058	0.954
Triple negative	0.171	1.19 (1.08–1.30)	0.047	3.614	< 0.001
Patient medical history					
History of disease	NA	1.00 (reference)	NA	NA	NA
Non-oncological medical history	0.258	1.29 (1.23–1.37)	0.028	9.339	< 0.001
Oncological medical history	1.612	5.01 (4.01–6.26)	0.114	14.198	< 0.001
Tumor stage					
Stage I tumor	NA	1.00 (reference)	NA	NA	NA
Stage II tumor	1.231	3.42 (3.33–3.53)	0.015	82.180	< 0.001

*NA* not applicable, *OR* odds ratio, *CI* confidence interval, *SES* socioeconomic status, *ILC* invasive lobular carcinoma

## Discussion

In this population-based study in a country where everyone has equal access to care, patients with newly diagnosed stage I or II breast cancer and patients with higher SES were significantly more likely to undergo BCT than mastectomy. SES did not affect whether-or-not patients underwent surgery-or-not, but older and higher tumor stage patients were less likely to undergo a surgical procedure. Furthermore, more BCT and fewer mastectomies are performed as the years go by.

The current study shows that for newly diagnosed stage I or II breast cancer patients, the higher the SES, the more likely it is that patients will undergo BCT, even in a country with equal access to care. This is in line with previous studies, regardless of whether there is universal healthcare or not [17–19]. Additionally, hospital with a lower breast cancer treatment volume was less likely to perform BCT. This is in line with previous studies which reported that hospital volume affects different aspects of breast cancer treatment [7, 28]. Additionally, reasons for these differences can be the preference of the clinician, unequal spread of SES within the Netherlands, age of patient, tumor grade, tumor stage, and hormone receptor status [7, 8]. Furthermore, a recent study showed that breast cancer patients with high SES are more likely to undergo postmastectomy reconstruction than their lower SES counterparts [29].

The finding that SES did not play a role in whether-or-not patients underwent surgery is in contrast with countries with no universal healthcare systems, in which therapeutic choices are heavily influenced by income and health care insurance system [17, 18]. Age being the most important factor determining whether a patient underwent surgery is probably related to the fact that older patients are more likely to have co-morbidities with a higher risk of postoperative complications, leading to surgery less often being advised [30, 31]. This could also explain why newly diagnosed stage I or II breast cancer patients who did not undergo surgery were significantly older than patients who did undergo surgery (over 20 years on average).

Over time, more BCT procedures but fewer mastectomies were performed. This is probably due to increasingly favoring BCT over mastectomy with its higher complication rate along with serious cosmetic and psychological consequences, while have similar overall survival [6, 32, 33].

The current study shows that for new-onset stage I or II breast cancer patients, the higher the SES, the more likely patients will undergo BCT. Furthermore, when adjusting for age, tumor characteristics, and medical history, these differences remain. The current study shows that even in a country with equal access to care, SES does play a role in whether a patient receives BCT or mastectomy despite there being no differences in (cancer-free) survival [6, 32, 33]. This is in line with previous studies, regardless of whether there is universal income or not [17–19]. The relation between hospital volume and immediate breast reconstruction could relate to organizational factors, such as the live attendance of a plastic surgeon to the multidisciplinary meetings, which is a factor influencing the immediate breast reconstruction and is easier to organize in a hospital with a large volume [29, 34]. Additionally, reasons for these differences can be the preference of the clinician, unequal spread of SES within the Netherlands, age of patient, tumor grade, tumor stage, and hormone receptor status [7, 8]. Furthermore, a recent study showed that breast cancer patients with high SES are more likely to undergo postmastectomy reconstruction than their lower SES counterparts [29].

The present study has some limitations. Other factors, which are not recorded in the NCR, may also be determinants of surgery type, such as race, ethnicity, health literacy, social environment, language, internet access, and religion. However, these factors are known to be closely linked to SES [9, 35, 36]. Nevertheless, more research is warranted to study the possible effects of cultural background on whether patients are given the same choices regarding breast cancer treatment when there is equal access to healthcare. Additionally, NCR does not record co-morbidities, which could also affect the therapeutic choice on whether or not to operate stage I or II breast cancer patients. Secondly, however, the current study does show that higher SES breast cancer patients are more likely to undergo BCT. The NCR does not have information about how well-informed patients are about their treatment options and if and to what extent shared decision making took place. Therefore, it would be interesting to study whether there also exist differences in information provision to women diagnosed with breast cancer regarding treatment options (non-surgical treatment, BCT, or mastectomy) between hospitals and/or regions in the Netherlands and whether this is influenced by patients’ SES or SES related factors. Moreover, maybe less access to patient information (due to, e.g., language barrier, illiteracy, less access to internet) might contribute to the fact that patients with lower SES are less likely to undergo BCT when operated.

In conclusion, the current study shows that even in a country with equal access to healthcare, stage I or II breast cancer patients with lower SES were less likely to undergo BCT. Age and tumor stage, but not SES, were associated with undergoing surgery-or-not.
